# A Case of Sweet Syndrome Complicating Ulcerative Colitis Treated With Upadacitinib

**DOI:** 10.14309/crj.0000000000001904

**Published:** 2025-11-24

**Authors:** Sankirth Madabhushi, Sarah Dixon, May Dong, Nicole Lewandrowski, Zendee Elaba, Jessica St. John, Andrea Poisson Irani, Abbas Rupawala

**Affiliations:** 1 Department of Medicine, UMass Chan Medical School, Worcester, MA; 2 School of Medicine, University of Galway, Galway, Ireland; 3 Department of Pathology, UMass Chan Medical School, Worcester, MA; 4 Department of Dermatology, UMass Chan Medical School, Worcester, MA; 5 Division of Gastroenterology, UMass Chan Medical School, Worcester, MA

**Keywords:** Sweet syndrome, upadacitinib, ulcerative colitis, inflammatory bowel disease

## Abstract

Sweet syndrome, also known as acute febrile neutrophilic dermatosis, is a rare inflammatory skin condition characterized by the sudden onset of painful, erythematous papules and plaques. It has been associated with underlying inflammatory bowel disease. We present a 24-year-old man with recently diagnosed ulcerative colitis on infliximab and methotrexate who was diagnosed by biopsy with Sweet syndrome. The patient was treated with multiple trials of steroids and biologics until finally showing improvement with upadacitinib. This case highlights the importance of considering Sweet syndrome in ulcerative colitis patients with atypical skin manifestations and the first documented case of Sweet syndrome treated with upadacitinib.

## INTRODUCTION

Sweet syndrome (SS), also known as acute febrile neutrophilic dermatosis, is characterized by fever, leukocytosis, and violaceous tender skin lesions that may be idiopathic, drug-induced, or associated with an underlying condition, infection, or pregnancy.^1^ It involves an overproduction of neutrophils and has been linked to systemic diseases, including ulcerative colitis (UC).^1^ SS typically presents with tender, erythematous papules that may evolve into plaques, pustules, and nodules, often affecting the face, neck, and other areas, accompanied by fever and neutrophilia.^2^ Diagnosis is based on clinical and histological examination.^3^ Differentiating SS from similar dermatological conditions such as erythema nodosum, pustular psoriasis, and pyoderma gangrenosum is crucial, as treatment strategies differ. Systemic corticosteroids are the first-line treatment of SS but are generally avoided in erythema nodosum unless severe.^4^ Current treatments for SS include discontinuing potential triggers and initiating a systemic steroid taper if symptomatic.^1^ For severe or refractory cases, there is limited evidence in support of alternative treatments such as NSAIDs, dapsone, and biologic medications.^5^ Upadacitinib is a second-generation Janus kinase inhibitor (JAKi) approved in 2022 for the treatment of UC.^6^ There are no reports showing the efficacy of upadacitinib in treating SS. This case report emphasizes the timely diagnosis of cutaneous pathologies associated with inflammatory bowel disease (IBD) and remission with upadacitinib.

## CASE REPORT

**Figure 1.**
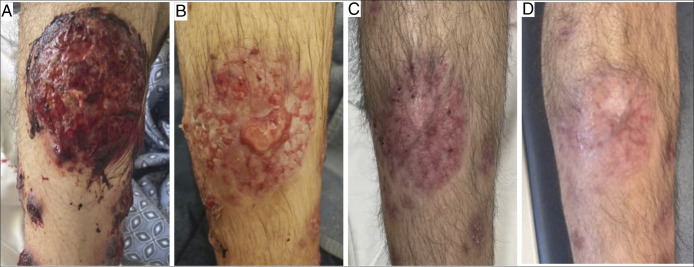
(A) August 2024–Violaceous ulcerated hemorrhagic plaque on right shin upon diagnosis. (B) October 2024–Right shin lesion improved since diagnosis, but showed evolving painful bullae while on ruxolitinib. (C) December 2024–Improvement in right shin lesion 1 month after starting upadacitinib. (D) February 2025–Continued improvement 3 months after starting upadacitinib.

**Figure 2.**
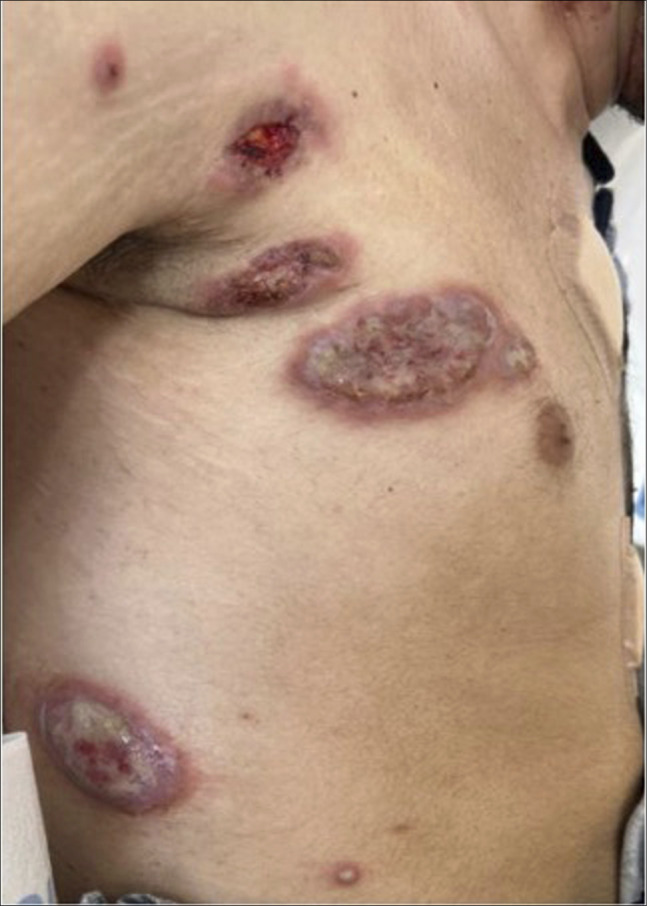
(A) August 2024–Multiple ulcerated erythematous pustular lesions on the right trunk/chest on diagnosis.

**Figure 3.**
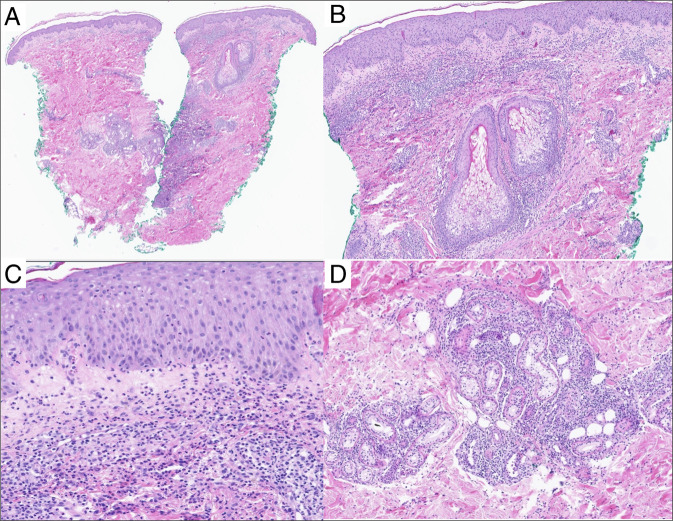
(A) Punch biopsy showing superficial and deep inflammatory infiltrate (hematoxylin-eosin, original magnification ×20). (B) Dermal edema is present (hematoxylin-eosin, original magnification ×40). (C) The inflammatory infiltrate is predominantly neutrophilic, with lymphocytes and histiocytes, in an interstitial and perivascular distribution (hematoxylin-eosin, original magnification ×100). (D) The infiltrate extends into the deeper dermis in a periadnexal distribution (hematoxylin-eosin, original magnification ×40).

**Figure 4.**
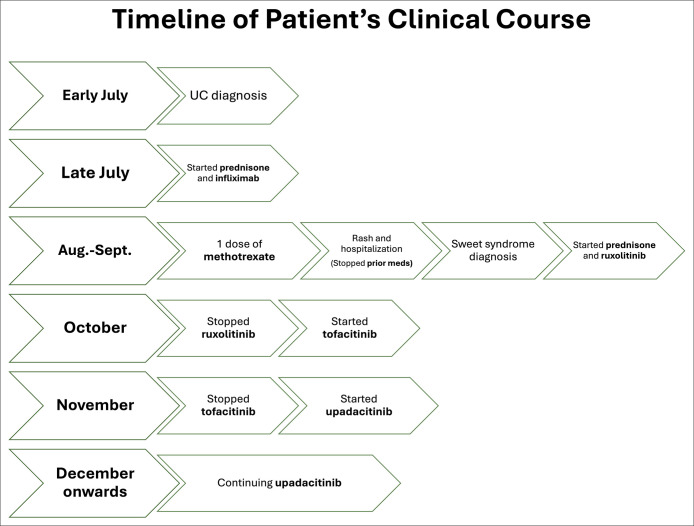
Timeline of patient's clinical course illustrating the chronological sequence of ulcerative colitis (UC) diagnosis, treatments, and the development of Sweet syndrome.

A 24-year-old man with recently diagnosed UC presented to the emergency department with one week of eruptive lesions on his scalp, face, neck, thorax, and extremities (Figures 1 and 2). He had biopsy-proven ulcerative pancolitis and initiated induction with systemic corticosteroids and infliximab one month before symptom onset and oral methotrexate 12.5 mg weekly one day before symptom onset. A broad infectious workup was negative. The painful, papular-pustular lesions rapidly increased in size and number, later ulcerating with purulent drainage, and a punch biopsy on day 2 of hospitalization showed interstitial granulomatous dermatitis with neutrophils, thus meeting the 2 major criteria for SS diagnosis (Table 1, Figure 3).^2^ In addition, the patient showed dramatic improvement within 24 hours of starting prednisone at 1 mg/kg. This excellent initial response to steroids, along with fever and a history of IBD, fulfilled 3 minor criteria for diagnosis (Table 1).^2^ On day 5, the infliximab trough level was 6.4 mcg/mL, and anti-drug antibodies were not detected. On discharge, the patient was prescribed a prednisone taper and ruxolitinib (Figure 1). One month after discharge, he continued to experience skin flares on ruxolitinib and was transitioned to another JAKi, tofacitinib. One month later, he continued to experience new skin lesions and worsening gastrointestinal symptoms, and he was transitioned to upadacitinib, another JAKi (Figure 1). One week later, he presented to the emergency department with a low-risk bilateral pulmonary embolism, but it was successfully treated inpatient with anticoagulation (Figure 4). Six months after starting upadacitinib, the patient had substantial improvement of his SS, with significant reduction in the pain, size, and ulceration of skin lesions, and his UC was in clinical remission (Figure 1).

**Table 1.** Diagnostic criteria for Sweet syndrome

Diagnostic criteria for Sweet syndrome^2^	
Criteria type	Criterion
Major criteria–*both* are required	1. Abrupt onset of painful, erythematous plaques or nodules
	2. Biopsy showing a dense neutrophilic infiltrate without evidence of leukocytoclastic vasculitis
Minor criteria—*at least 2* are required	3. Pyrexia >38 °C
	4. Association with: a. Malignancy b. Inflammatory disease c. Pregnancy d. Preceding respiratory or gastrointestinal infection e. Vaccination
	5. Good response to corticosteroids or potassium iodide
	6. At least 3 abnormal laboratory findings: a. Erythrocyte sedimentation rate > 20 mm/hr b. Elevated C-reactive protein c. White blood cell count >8,000/mm^3^ d. Neutrophils >70% on differential

## DISCUSSION

SS is often associated with underlying systemic conditions such as IBD. To date, there have been no reports documenting the efficacy of upadacitinib in the treatment of SS, making this the first reported case of its use in a patient with UC. This case highlights the importance of considering SS in UC patients with unusual skin manifestations, especially when there is concern for bacterial infection or in men, where the disease is less common. A timely biopsy can reduce delays in initiating corticosteroids, the first-line treatment for both UC and SS, and decrease unnecessary broad-spectrum antibiotic use.

This case highlights the diverse clinical presentations of SS, including large, black and red pyoderma-like ulcers and hemorrhagic papules and plaques while on infliximab and methotrexate. The patient had a therapeutic trough level, lacked anti-drug antibodies, and his UC was responding to infliximab during the treatment induction phase, but infliximab was discontinued on the onset of his rash of unclear etiology. The 3 recognized types of SS are classical/idiopathic, malignancy-associated, and drug-induced.^1^ Classical/idiopathic is often associated with IBD, upper respiratory tract infection, and gastrointestinal infection. This patient's presentation is most consistent with the classical form secondary to UC. A malignancy workup was performed to evaluate for malignancy-associated subtypes that may be seen with hematopoietic or solid cancers, with the former being more common. The workup included complete blood counts and chest computed tomography (CT) and abdominal/pelvic CT scans, which showed no evidence of malignancy. A flexible sigmoidoscopy performed one month before admission showed findings consistent with UC, with biopsies revealing no evidence of malignancy. A complete colonoscopy was planned to further evaluate for underlying disease in the future. The drug-induced form was also considered. This type is triggered most often by antineoplastic agents though, and the symptoms typically disappear after the drug is stopped. The patient did not have any home medications besides infliximab and methotrexate. Previous case reports have noted the occurrence of SS in patients receiving combination therapy with infliximab and azathioprine or with adalimumab in the context of IBD, but the evidence supporting these associations remains limited.^7–9^ Infliximab-induced pustular psoriasis, which can present with widespread, tender erythematous patches studded with numerous pinhead-sized pustules, was considered in the differential diagnosis but was excluded on the basis of biopsy findings.^10^

While the exact mechanism is incompletely understood, upadacitinib may treat SS associated with IBD by targeting the Janus kinase (JAK)-signal transducer and activator of transcription signaling pathway implicated in its pathogenesis.^11–13^ Targeting this pathway may reduce the activity of proinflammatory cytokines and neutrophils consistently observed in SS lesions and in IBD flares.^11–13^ Upadacitinib inhibits cytokines that use JAK1-containing receptor complexes, such as interferons, interleukin -6, and γ-chain cytokines.^14,15^ Conversely, tofacitinib is a pan-JAK inhibitor that affects multiple JAK isoforms, including JAK1, JAK2, and JAK3.^14,15^ Tofacitinib targets the same cytokines as upadacitinib, along with others such as granulocyte colony-stimulating factor (G-CSF) and interleukin-3, potentially leading to broader immunosuppressive effects.^14^ The selectivity of upadacitinib for JAK1 may offer a more focused anti-inflammatory effect, minimizing off-target effects while effectively disrupting the inflammatory cascade central to both SS and IBD.^14,16^

In addition, a multidisciplinary approach tailored to this patient's needs was instrumental in treatment. Although prednisone provided initial relief, the sheer severity of his lesions and active UC warranted an aggressive second-line therapy, with a JAKi to manage both gastrointestinal and dermatologic conditions. While on ruxolitinib, the patient continued to have multiple episodes of diarrhea throughout the day and night, and he developed a new skin lesion. After extensive discussions between dermatology and gastroenterology, upadacitinib was prescribed. While dapsone or colchicine may treat SS, there is a lack of high-quality clinical trials or guideline recommendations for their use in UC.^5^ We avoided using NSAIDs because of worsening skin lesions on therapy and concern for aggravating UC.^17^

The patient was also evaluated by hematology after his pulmonary embolism. The patient had self-discontinued tofacitinib 2 weeks before the event and had started upadacitinib one week before. It was noted that postmarketing trials of tofacitinib had shown an increased risk of venous thromboembolism (VTE).^18^ However, data on VTE with JAKi in general are mixed, with boxed warnings based largely on postmarketing surveillance and pharmacovigilance studies.^19,20^ The risk of VTE is not definitively established for other JAKi, and recent meta-analyses have shown no statistically significant increase in the thromboembolic risk of upadacitinib compared with a placebo.^21,22^ Moreover, it was our clinical impression that the VTE was a result of his uncontrolled inflammation and long duration of high-dose steroid use, which increases the risk for VTE in the setting of severe UC. Thus, the benefits of continuing upadacitinib for his severe diseases outweigh the uncertain risk of VTE. In short, upadacitinib can be considered for the treatment of severe SS in patients with underlying IBD.

## DISCLOSURES

Author contributions: S. Madabhushi wrote and edited the article and is the article guarantor. S. Dixon, and M. Dong assisted in writing various portions of the case report and creating the figures, respectively. N. Lewandrowski and Z. Elaba wrote about the histopathologic findings in the case. J. St. John provided dermatologic expertise for reporting this case. A. Poisson Irani, and A. Rupawala oversaw the team and provided additional guidance.

Financial disclosure: None to report.

Previous presentation: 2025 Crohns and Colitis Congress Poster Presentation, Sankirth Madabhushi (Presenting Author); February 6, 2025; San Francisco, California.

Informed consent was obtained for this case report on April 20, 2025.

## ABBREVIATIONS:

CT: computed tomography
G-CSF: granulocyte colony-stimulating factor
IBD: inflammatory bowel disease
JAK: Janus kinase
JAKi: Janus kinase inhibitor
SS: sweet syndrome
UC: ulcerative colitis
VTE: venous thromboembolism
